# Successful Use of Immunotherapy in a Patient with Metastatic Squamous Cell Lung Cancer and Underlying Autoimmune Disease

**DOI:** 10.7759/cureus.15918

**Published:** 2021-06-25

**Authors:** Lusine Zakharian, Lauren Lee

**Affiliations:** 1 Internal Medicine, Brooke Army Medical Center, Fort Sam Houston, USA; 2 Hematology and Oncology, Brooke Army Medical Center, Fort Sam Houston, USA

**Keywords:** immunotherapy, immune, check-point, lupus, adverse, metastatic, lung, cancer, pembrolizumab, autoimmune

## Abstract

The immune system removes abnormal and cancerous cells by way of T-cell detection at immune checkpoints. Cancerous cells, due to their expression of proteins such as T-cell inactivating programmed death-ligand 1 (PD-L1), may evade the immune system resulting in replication and ultimately metastases. Immunotherapy in the form of checkpoint blockade, such as anti-programmed cell death 1 (PD-1) monoclonal antibody pembrolizumab, targets and interferes with this interaction, thereby restoring T-cell ability to remove cancer cells. Immunotherapy has revolutionized cancer treatment and has improved survival in several malignancies. However, the presence of autoimmune disease is an exclusion criterion for most immunotherapy trials due to fear of potentially life-threatening immune system activation. Therefore, its safety and efficacy in patients with autoimmune disease are not well studied. We describe the successful use of pembrolizumab in a patient with systemic lupus erythematosus (SLE) and review available literature, demonstrating that there is a subset of patients with underlying autoimmune disease who can safely be treated with immunotherapy. Furthermore, that administration of traditional cytotoxic chemotherapy prior to immunotherapy may lead to autoimmune disease control by eliminating autoantibodies.

## Introduction

The immune system is the first line of defense against malignancies. Immune system function in combatting malignancies involves T-cells which detect cancer cells at immune checkpoints, subsequently inducing cancer cell death. Pembrolizumab is a type of immunotherapy known as a checkpoint inhibitor; it is an anti-programmed cell death 1 (PD-1) monoclonal antibody. Programmed death-ligand 1 (PD-L1) is a protein that cancer cells may express which allows these cells to bind to T-cell PD-1 and B7 proteins at an immune checkpoint, allowing for evasion of T-cell induced apoptosis of these cancer cells, leading to replication and ultimately metastases. Pembrolizumab interferes with this interaction by restoring T-cell function, allowing for removal of cancerous cells. In metastatic squamous cell lung cancer, immunotherapy has dramatically improved patient outcomes and survival [1]. However, the presence of autoimmune disease is a relative contraindication for the use of checkpoint inhibitors due to potentially life-threatening immune toxicities and activation of the underlying autoimmune condition [2,3]. We describe a unique case of a patient with systemic lupus erythematosus (SLE) and metastatic squamous cell lung cancer who failed treatment with traditional cytotoxic chemotherapy but has subsequently been successfully treated with pembrolizumab for greater than 18 months without any severe immune-related adverse events or SLE activation.

## Case presentation

**Figure 1:**
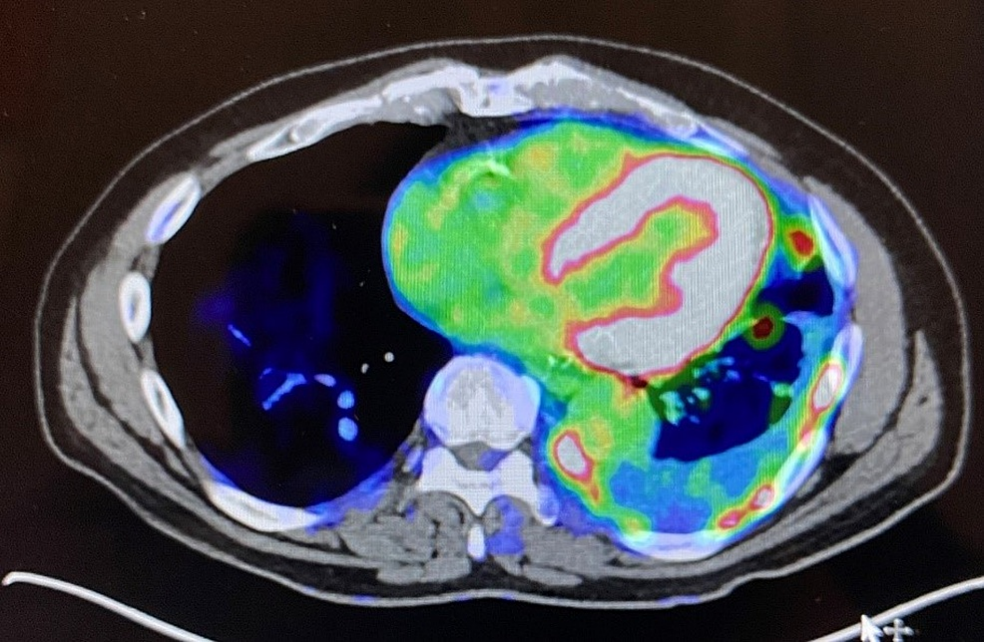
Hypermetabolic 3.4-centimeter mass with left pleural thickening and associated lytic rib lesions.

**Figure 2:**
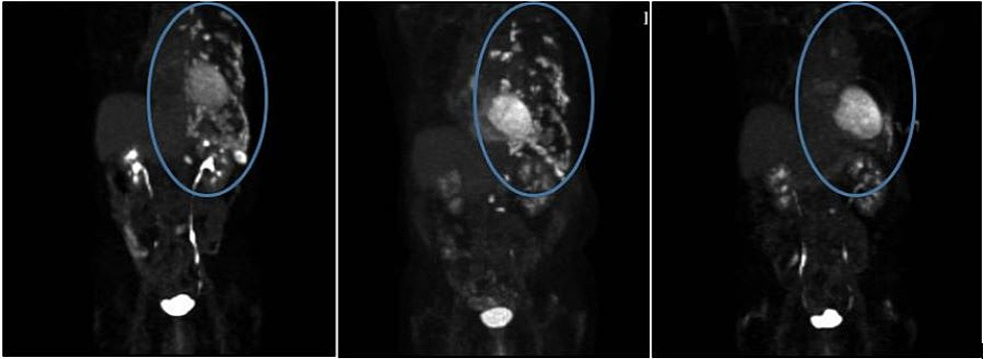
Image on left showing PET/CT at diagnosis, middle image with PET at time of progression, and right image demonstrating PET with partial response.

A 68-year-old man with a long-standing history of SLE with associated discoid lesions and high-titer antiphospholipid antibodies (APLAs), presented with severe left rib pain, anemia, and hypercalcemia. PET imaging revealed a hypermetabolic 3.4-centimeter mass with left pleural thickening and associated lytic rib lesions, left upper lobe nodule, and hypermetabolic periaortic, left hilar, and mediastinal lymph nodes suspicious for metastasis (Figure 1). Tissue biopsy revealed metastatic squamous cell carcinoma of the lung with PD-L1 immunohistochemical analysis tumor proportion score (TPS) of 95%. Secondary to concern for activation of the patient’s lupus and risk for catastrophic thrombosis with high-titer APLAs, immunotherapy was avoided despite high PD-L1 expression. Traditional cytotoxic chemotherapy with carboplatin and nab-paclitaxel was initiated. While this initially led to a partial response, his cancer eventually progressed resulting in his inability to perform daily tasks, severe weight loss, and marked pain, ultimately culminating in emergency room presentation with a large left pleural effusion requiring a pleural catheter and subsequent drain placement. In light of his well-controlled lupus not requiring concomitant immunosuppressive therapy, in conjunction with his severe symptoms, and limited treatment options, the decision was made to proceed with palliative pembrolizumab. Within a few weeks, he experienced decreased pain, drain removal, and increased mobility. Within months, he achieved partial remission with excellent response and complete resolution of symptoms (Figure 2). Throughout his treatment, he has had no severe SLE flares or thrombosis. A mild flare in discoid lesions was successfully treated with local corticosteroids. After treatment with pembrolizumab for greater than 18 months, he has had dramatic improvement in quality of life, clinical symptoms, and full return to function.

## Discussion

The current standard of care for patients diagnosed with metastatic squamous cell carcinoma of the lung consists of chemotherapy and/or immunotherapy. If tumor PD-L1 expression is greater than 50%, treatment with immunotherapy alone is recommended. This immune system activation, however, can lead to severe immune-related adverse events such as life-threatening autoimmune colitis, hepatitis, and pneumonitis [2]. Therefore, patients with pre-existing inflammatory or autoimmune conditions have been largely excluded from immunotherapy clinical trials. As a result, it is a relative contraindication to use immunotherapy in this patient population, particularly in those actively receiving immunosuppressive therapy [3]. Appropriately, immunotherapy was initially avoided due to concern for immune-related side effects and catastrophic thrombosis given the patient’s underlying SLE and high titer APLAs. However, given his toxic course and continued progression of disease, immunotherapy was trialed with recognition of potential SLE reactivation. This case highlights that immunotherapy can be used safely in a subset of patients with autoimmune conditions. Furthermore, that chemotherapy first, which may be cytotoxic to cells producing antibodies responsible for SLE, or in combination with immunotherapy can be safely done with close monitoring.

Pembrolizumab is an anti-PD-1 monoclonal antibody, also known as a checkpoint inhibitor. PD-L1 is a protein that cancer cells may express which allows these cells to bind to T-cell PD-1 and B7 proteins, bypassing an immune checkpoint, allowing for evasion of T-cell induced apoptosis of cancer cells. Pembrolizumab interferes with this interaction, restoring T-cell function. Initiating immune checkpoint inhibition in patients with underlying autoimmune disease is reasonably approached with caution given the available literature. In a case series by Coureau et al., 37 publications reported 41 cases of patients with pre-existing autoimmune disease; in most cases (n = 29, 65.6%) immunotherapy resulted in a flare of the baseline disease which required treatment with steroids, infliximab, adalimumab, or rituximab. In 10 cases (22.7%), flares were considered severe/very severe, with one death: a flare of infliximab-resistant colitis with progression to toxic epidermal necrosis [4]. Another notable and severe case was the development of type one diabetes mellitus presenting with diabetic ketoacidosis after one dose of pembrolizumab, without any previous diagnosis of autoimmune disease [5]. Suggesting that perhaps in the right host, immunotherapy may even trigger a previously dormant autoimmune condition. Similarly, a case of SLE onset is seemingly induced and associated with a patient receiving pembrolizumab [6]. Therefore, there is evidence to suggest that patients with underlying autoimmune disease have appropriately been excluded from immune checkpoint inhibitor clinical trials. It is important to note, however, that in most of these cases (90%), patients were successfully treated with steroids or another immunosuppressive agent [4]. In the metastatic setting where treatment options are limited and there is close monitoring with immunosuppressants readily available, it is rational to initiate immunotherapy. Use of immunotherapy in the adjuvant setting, where risks may outweigh benefits, is less clear cut. At this time there are no specific guidelines for the type of underlying autoimmune disease, or its severity, that may be reasonably considered for immunotherapy treatment. If an autoimmune flare can be easily treated with local therapy or a short course of low-dose steroids, the benefit of immunotherapy likely outweighs the risks. Furthermore, if the flare does not pose organ-threatening manifestations (i.e., small rash or joint pain versus nephritis or pericarditis), use of immunotherapy is more justified.

While patients with autoimmune disease have been intentionally excluded from clinical trials, there are cases where patients with autoimmune disease have been included in clinical trials unintentionally. Results of these do provide some additional safety evidence. The FDA completed a post-hoc analysis based on aggregate data with four anti-PD-1/PD-L1 agents. In total, 552 patients enrolled in 22 clinical trials of PD-1/PD-L1 inhibitors were identified with a history of autoimmune disease. None were known to be dependent on systemic corticosteroids at baseline. Worsening of underlying autoimmune disease occurred in 6%-16% of cases, with higher occurrence with longer duration of checkpoint inhibitor use [7]. In this population, PD-1/PD-L1 inhibitors seem safe given the low incidence of flares. In addition to this, there were no reported deaths, and patients were successfully treated for flares when they occurred. It should be noted, however, that there may be a bias toward mild autoimmune disease given that none of the patients were dependent on systemic corticosteroids at baseline. A cohort study by Van der Kooij et al. further demonstrated that immune-related adverse events were similar between patients with and without underlying autoimmune disease who were treated for advanced melanoma with immune checkpoint inhibitors [8]. Importantly, they did report that patients with inflammatory bowel disease were more likely to develop severe colitis and toxicity requiring early discontinuation of treatment. Therefore, data on severity and treatment of baseline disease in these analyses is not clear. However, it is reasonable to consider that patients with mild autoimmune disease may tolerate immunotherapy well.

Our case also raises the possibility that chemotherapy first, prior to immunotherapy, may play a role in enhancing the safety of immunotherapy treatment by reducing antibodies responsible for autoimmune disease. Considerable evidence shows that combined chemotherapy and pembrolizumab for the treatment of metastatic non-small cell lung cancer results in increased progression-free disease and improvement in overall survival [9]. However, the effect of various sequences remains unclear. With our patient, it seems that the quiescence of SLE may be associated with the fact that chemotherapy was given first. On presentation, he had high-titer APLAs. After chemotherapy, these were undetectable and no thromboses occurred throughout his treatment (Table 1). Chemotherapy agents such as methotrexate and cyclophosphamide are commonly used and part of treatment algorithms for various autoimmune diseases [10,11]. Similar cytotoxic mechanisms of immune cell death are seen with gemcitabine and paclitaxel. Therefore, it not unreasonable to suggest that treatment with initial chemotherapy potentially eliminated our patient’s SLE-associated antibodies. This is an area of necessary research that may enhance our understanding of the safety and risk stratification of immunotherapy for patients with cancer and underlying autoimmune disease.

**Table 1:** Labs dated 10/03/2018 and 11/05/2019, respectfully. Lupus anticoagulant undetectable after chemotherapy.

Lupus Anticoagulant Panel	Units	Ref Range	Lupus Anticoagulant Panel	Units	Ref Range
Lupus Anticoagulant Neutralization High Phospholipid	10.9 (H) Sec	(0.0-7.9)	Lupus Anticoagulant Neutralization High Phospholipid	0.0	(0.0-7.9)

## Conclusions

Patients with underlying autoimmune disease have generally been excluded from immunotherapy clinical trials. Analysis of past patient databases, however, seems to favor overall safety of immunotherapy in select patients, such as those with mild disease or disease not involving critical organs. Furthermore, the optimal sequence of cytotoxic chemotherapy and immunotherapy is unclear, but it is possible that cytotoxic therapy may have simultaneously treated our patient’s SLE. This case highlights that there is a subset of patients with underlying autoimmune disease that can safely and efficaciously be treated with immunotherapy and that chemotherapy first may provide protection from immune-related adverse events. We propose that patients with autoimmune disease with minimal organ involvement who are not on active immunosuppression may safely be treated with immunotherapy and urge their inclusion in immunotherapy clinical trials.
